# The role of biostatistics in the response to COVID-19: a Belgian and international perspective

**DOI:** 10.1186/s13584-023-00554-z

**Published:** 2023-01-31

**Authors:** Geert Molenberghs

**Affiliations:** I-BioStat, Universiteit Hasselt and KU Leuven, Hasselt, Belgium

**Keywords:** Data availability, Data sharing, Communication, Advisory bodies

## Abstract

In this commentary to Dattner et al*.* (Israel J Health Policy Res. 11:22, 2022), we highlight similarities and differences in the role that biostatistics and biostatisticians have been playing in the COVID-19 response in Belgium and Israel. We bring out implications and opportunities for our field and for science. We argue that biostatistics has an important place in the multidisciplinary COVID-19 response, in terms of research, policy advice, and science and public communication. In Belgium, biostatisticians located in various institutes, collaborated with epidemiologists, vaccinologists, infectiologists, immunologists, social scientists, and government policy makers to provide rapid and science-informed policy advice. Biostatisticians, who can easily be mobilized to work together in pandemic response, also played a role in public communication.

## Commentary

In an important article [1], the role of statisticians in the response to COVID-19 in Israel is discussed. The authors produced a clear and fair account of contributions made and opportunities missed by (bio)statisticians. Documenting a country’s or a region’s experience is a valuable contribution to pandemic preparedness. Such documents can serve as input for national and international debate. Interaction between countries, even of an informal nature, is particularly useful [2]. We focus on the importance of pre-existing structures, research, policy advice, communication, lessons learned, and data availability. The role of biostatisticians is threefold: in research, policy advice, and communication. Starting from my own experience in science and public communication and as a member of various government advisory bodies during the pandemic, I will highlight the role played by biostatistics and biostatisticians in Belgium. The field played a prominent role, alongside epidemiology, virology, immunology, infectiology, and the social and behavioral sciences. There was a close collaboration across academia, government public health agencies, and policy makers. Because of pre-existing structures (e.g., learned societies) and collaborations, biostatisticians working in various entities can rapidly join efforts.

## Pre-existing structures

Biostatisticians at the Belgian universities of Hasselt and Leuven have had a long- standing collaboration that materialized in a joint research institute (I-BioStat; Interuniversity Institute of Biostatistics and statistical Bioinformatics). There is also a three decade old collaboration between the universities of Hasselt and Antwerp in infectious diseases with focus on mathematical modeling via the SIMID consortium (Simulation Models of Infectious Diseases) (https://www.simid.be). It extends beyond this theme to research in vaccines, antiviral medication, health economic evaluation, etc. This collaborative effort involves biostatisticians, epidemiologists, vaccinologists, microbiologists, virologists, data scientists, etc. As noted by Dattner et al*.* [1], such pre-existing collaborations are of great value. In Belgium, for example, it has led to a broad-based article on the transformative effect of the pandemic on biostatistical, epidemiological, and clinical research [3].

The critical mass of I-BioStat, and its link to statisticians in other disciplines allowed us in March–April 2020 to extend capacity to enable both a rapid and long-term pandemic response in difficult circumstances.

In Belgium, like elsewhere, there is capacity in academia, government, and industry. In Recommendation 2 of Dattner [1], capacity that cuts across academia and industry is suggested, but I would like to add government as well. Conflicts of interest need to be carefully assessed (e.g., colleagues working on vaccine development in the industry). That said, biostatisticians, wherever located, will typically be able to quickly transition to pandemic response work. Evidently, government health and official statistics institutes will harbor relevant personnel. For successful pandemic response, multidisciplinary collaboration is key, in an atmosphere of competence, respect, and trust.

As Dattner et al*.* [1] states, competences needed change over time in such a long health crisis. This requires a large and flexible network. An interesting example is Israel’s vaccine effectiveness research, and research on the waning of effectiveness during the fourth wave. Given that Israel was ahead of many countries in its vaccination campaign, the work was relevant nationally and internationally.

## Research

Research is the backbone of our scientific authority. A centerpiece is mathematical modeling, supplemented with competence in longitudinal data, time series, incomplete data, spatial methodology, general statistical modeling, forecasting and nowcasting, artificial intelligence, machine learning, clinical trial methodology, etc. A successful network should encompass many of these.

## Policy advice

The Belgian pre-existing biostatistics and infectious diseases epidemiology hub was known to policy makers, so they called upon colleagues from Hasselt University from the start, for scientific and policy advice work.

Policy advisory boards are diverse: some continually exist but are dormant when there is no health crisis, while others are ad hoc. In Belgium, the advisory structure changed several times throughout the pandemic, in part because there was a change in federal government in late-2020. Some uncluttering is needed in the advisory board structure; even though a health crisis is complex, the overall structure should be simple and transparent [2].

Unlike in Israel, Belgian biostatisticians and epidemiologists have always been present on advisory boards. As in many countries, there has been a continual debate, also in academic circles, as to whether the advisory boards were sufficiently balanced and representative, even though virologists, epidemiologists, and biostatisticians worked alongside economists, sociologists, psychologists, immunologists, pediatricians, etc. Such exposure is virtually absent in non-pandemic times and thus we may not be fully prepared for the challenges posed by pandemics [4].

## Communication

Experts are needed not only to provide policy advice, but also to communicate with the media and the general public and explain key concepts. Early in the pandemic, concepts such as exponential growth, mitigation strategies, and the evidence for non-pharmaceutical interventions needed to be explained. Training in science communication and interaction with the media are needed[1]. The epidemiology of infectious diseases is complex, especially with a pathogen that itself is poorly understood in its early days. Yet, clear and simple—though scientifically accurate -communication is needed. Statisticians are best placed to communicate about “signal and noise,” not just signal. We have been confronted by the media and a public that are looking for certainty when none can be given. The fact that the population’s own behavior is able to alter the course of the pandemic, modulated by government policy and public trust in government, implies that science cannot be an oracle of exact predictions. Biostatistics can contribute a careful quantitative assessment of what has been happening and sketch how the epidemic will evolve over the coming period under various scenarios.

As the pandemic unfolded, increasingly critical voices were heard, in academia, politics, media, and the general public. While healthy, there comes a point where genuine debate is overshadowed by conspiracy theories, hate messages, and even threats to researchers’ mental and physical well-being, sometimes necessitating police protection and other protective measures. The Royal Netherlands Academy of Arts and Sciences [4] has examined the consequences of this and formulated recommendations to empower and protect scientists in the spotlight, and to protect scientific debate and the scientific method. Individual scientists should know the limits of their expertise and try not to opinionate outside of it. Multidisciplinary advisory bodies and scientific networks can help overcome this potential pitfall.

Scientists who offer policy advice and communicate publicly should understand the tension involved in bringing a clear yet not overly alarming message to the general public, while conveying a sense of urgency to policy makers.

## Learn the lessons that can be learned

It is crucial to document the various scientific groups’ experiences with pandemic response, in particular the statisticians’, so that notes can be compared, good practices copied, and replication of errors avoided.

Another key lesson is that in many parts of the world the pandemic response has been too geographically uncoordinated. This is true in regions with strongly inter-connected countries, such as in Europe, especially in the Schengen zone. Disparate measures in trans-border regions simply lead to adverse effects (e.g., closing shops in one country, but not in the neighboring one, induces more rather than less mobility). Ideally, identical or at least similar measures would be taken in homogeneous regions, whether or not they cross national borders.

Even with more isolated countries (e.g., island nations), maintaining formal and informal links is crucial. Evidently, we have dedicated international health structures, such as WHO and its regions, ECDC, etc. that can play important roles. At the same time, our formal (learned societies) and informal networks of people that share the same scientific language are also a valuable resource.

As outlined in Molenberghs [3], the pandemic has induced an unprecedented speed in the development of vaccines and antivirals. It may suggest simplification of procedures in non-pandemic times as well. Clinical trials should not be carried out in a silo; instead, drug development should interact with “real world data/evidence”, closing the gap between biostatistics and quantitative epidemiology.

## Conclusions

It is crucial for statisticians to be embedded in a strong and active network that is able to rapidly respond to a health crisis. Evidently, networks should bring together biostatisticians from various academic and government research institutes, but they should also encompass researchers from other fields, in particular epidemiology, virology, immunology, infectiology, and the social and behavioral sciences. Such a network should have ready access to all the necessary data, preferably in real time. The data flow should be properly organized prior to a health crisis, so that no valuable time is lost. Statisticians as well as other scientists should be able to take up their role in a multi-disciplinary effort to provide policy advice and engage in science communication.

## Data Availability

I fully agree with Dattner et al*.* [1] that high-quality data repositories are of paramount importance. When confronted with a nascent pandemic, with each new wave, the growth of yet another variant-of-concern, and the evolving vaccination campaigns, good data are needed. Speed trumps perfection, but practice may be different. Proprietary and privacy issues may take too much time. The research on vaccine waning in Israel was an example of what is possible. Other examples are the high-quality reports of the United Kingdom’s UKHSA and the Danish Statens Serum Institut (https://en.ssi.dk). This mattered when both of these countries were ahead of most others in the spread of the first omicron variant BA.1. The sophistication of the statistical and mathematical models is very important, but they are of no use without good data.
